# Burden of disease in myasthenia gravis: taking the patient’s perspective

**DOI:** 10.1007/s00415-021-10891-1

**Published:** 2021-11-20

**Authors:** Sophie Lehnerer, Jonas Jacobi, Ralph Schilling, Ulrike Grittner, Derin Marbin, Lea Gerischer, Frauke Stascheit, Maike Krause, Sarah Hoffmann, Andreas Meisel

**Affiliations:** 1 Department of Neurology with Experimental Neurology, Charité University Medicine Berlin, Corporate Member of Freie Universität Berlin and Humboldt-Universität Zu Berlin, Charitéplatz 1, 10117 Berlin, Germany; 2 NeuroCure Clinical Research Center, Charité University Medicine Berlin, Charitéplatz 1, 10117 Berlin, Germany; 3 Center for Stroke Research Berlin, Charité University Medicine Berlin, Charitéplatz 1, 10117 Berlin, Germany; 4 Institute of Biometry and Clinical Epidemiology, Charité University Medicine Berlin, Charitéplatz 1, 10117 Berlin, Germany; 5 Institute for Social Medicine, Epidemiology and Health Economics, Charité University Medicine Berlin, Luisenstraße 57, 10117 Berlin, Germany; 6 Core Facility Genomics, Berlin Institute of Health at Charité University Medicine Berlin, Charitéplatz 1, 10117 Berlin, Germany; 7 Department of Psychiatry, Psychotherapy and Psychosomatics, Charité University Medicine Berlin at St. Hedwig Hospital, Große Hamburger Straße 5-11, 10115 Berlin, Germany

**Keywords:** Myasthenia gravis, Quality of life, Burden of disease, Real-world setting

## Abstract

**Background:**

Myasthenia gravis (MG) leads to exertion-dependent muscle weakness, but also psychological and social well-being are limited. We aim to describe the burden of disease in MG including sociodemographic, economical, psychosocial as well as clinical aspects, to compare health-related quality of life (HRQoL) of patients with MG to the general population (genP) and to explore risk factors for a lower HRQoL.

**Methods:**

This case–control study was conducted with MG patients of the German Myasthenia Association. A questionnaire-based survey included sociodemographic and clinical data as well as standardized questionnaires, e.g. the Short Form Health (SF-36). HRQoL was compared to genP in a matched-pairs analysis. Participants of the German Health Interview and Examination Survey for Adults (DEGS1) served as control group.

**Results:**

In our study, 1660 MG patients participated and were compared to 2556 controls from the genP. Patients with MG showed lower levels of physical functioning (SF-36 mean 56.0, SD 30.3) compared to the genP (mean 81.8, SD 22.1, adjusted difference: 25, 95% CI 22–29) and lower mental health sub-score (SF-36 mean 67.3, SD 19.8, vs. 74.1, SD 16.7, adjusted difference: 5, 95% CI 2–8). Female gender, higher age, low income, partnership status, lower activities of daily life, symptoms of depression, anxiety and fatigue and self-perceived low social support were associated with a lower HRQoL in MG patients.

**Discussion:**

HRQoL is lower in patients with MG compared to genP. The burden of MG on patients includes economic and social aspects as well as their emotional well-being. New therapies must achieve improvements for patients in these areas.

**Trial registration information:**

Clinicaltrials.gov, NCT03979521, submitted: June 7, 2019, first patient enrolled: May 1, 2019, https://clinicaltrials.gov/ct2/show/NCT03979521

**Supplementary Information:**

The online version contains supplementary material available at 10.1007/s00415-021-10891-1.

## Background

Myasthenia gravis (MG) is a rare autoimmune disease with a prevalence of 15–20/100,000 inhabitants [12]. First symptoms appear with an age peak around 30 and 70–80 years of age [1]. Specific antibodies affect the neuromuscular junction and lead to fluctuating fatigability and weakness of the ocular, bulbar and skeletal muscles. In 15%, no antibodies can be detected (i.e. seronegative) [12]. Therapy with acetylcholinesterase inhibitors, immunosuppressive agents and thymectomy, lead to a stable condition in most patients with only mild to moderate motor symptoms. Despite this, an estimated 10–20% of patients with MG do not achieve an adequate response or are intolerant to conventional treatment [43]. These refractory cases concern more often females and are typically younger at disease onset [29]. New therapeutic strategies like the complement-inhibitor eculizumab [18] have been developed and several more are in the pipeline.

Apart from motor symptoms, also psychological and social well-being are limited in patients with MG [17]. The increasing interest in health-related quality of life (HRQoL) in MG patients is reflected by a growing number of studies in this field. Tools as the widely recognized SF-36 questionnaire and the MG-specific MG-QoL15 have been used to measure the HRQoL in several MG cohorts [2, 4–6, 23, 31, 37–39, 44, 51]. The studies show consistently that severe muscle symptoms and disability are associated with lower physical scores of HRQoL [37, 38, 51]. Symptoms of depression frequently affect the HRQoL negatively [23, 44, 51]. Patient characteristics, such as gender, age, education, course of disease, the use of immunosuppressive drugs, the occurrence of side effects, acceptance of disease as well as anxiety and perceived social support, have been demonstrated to be additionally associated with a poor quality of life in MG patients [2, 5, 45]. However, none of these studies determined whether the factors influencing HRQoL are myasthenia-specific or also apply to the normal population.

The so-called global *burden of disease* is a concept that was developed in the 1990s in a cooperation with the World Health Organization (WHO) to describe death and loss of health due to diseases, injuries and risk factors for all regions of the world. The gap between an ideal situation, where everyone lives free of disease and disability, and the cumulated current health status, is defined as the burden of disease [16]. So far, the *burden of disease* in MG in particular and its specific risk factors are not well defined. In treatment-refractory patients, factors like disability, drug- or surgery-associated adverse events, myasthenic crises, MG-related hospitalization, and comorbidities indicate a high burden [6]. Further, unemployment, lower mental health and HRQoL are likely to be associated with a high burden in treatment-refractory patients [43]. So far, the burden of disease in *non*-refractory patients has not been described.

The aim of this study is to estimate the burden of MG based on a representative patient population using a multidimensional approach. In a case–control study, we matched MG patients with the general population (genP) to compare the HRQoL and to explore myasthenia-specific risk factors for a lower HRQoL.

## Methods

### Data collection

In May 2019, the 3262 members of the German Myasthenia Association (Deutsche Myasthenie Gesellschaft, DMG) received the study information and a questionnaire as well as a pre-stamped envelope addressed to the coordinating study centre. The study participants (SP) were instructed to return their completed questionnaire without any further identifying information to ensure the anonymity of the survey. No refund was given. Returned questionnaires were accepted within the cut-off date of 31 July 2019.

### Questionnaire

The questionnaire concerned demographic data (gender, age, marital status/partnership, size of family), educational status, employment, income, and possession of a severely disabled person card (in Germany delivered at a certain degree of disability ranging from 10 (mild) to 100 (very severe)) were asked.

Regarding the medical aspects of MG, the questionnaire asked for age at symptom onset, age at medical diagnosis, subtype (ocular versus generalized), antibody (Abs) status (Acetylcholine receptor–antibody (Ach-R-Abs), muscle-specific kinase antibody (Musk-Abs), (Lipoprotein-related protein 4 antibody (LRP4-Abs), seronegative), comorbidities, thymectomy, current MG-specific medication (cholinesterase inhibitors, glucocorticoids, long-term immuno-suppressants, monoclonal antibodies, plasmapheresis (PE)/immuno-absorption (IA), intravenous immunoglobins (IVIG)) including dosage/frequency, co-medication (antidepressants, painkillers), side effects and treatment satisfaction.

Most questions were asked with a checkbox option, always specified to be answered as a single or multiple-choice option. Only few questions were asked as free-text format. The questionnaires were scanned and processed with the software TeleForm (OpenText), version 10.9.1.

### Definitions

In subgroup analysis, we defined patients with generalized MG, self-rated moderate or high disease severity and any exacerbation medication use in the past (IVIG, PE, Rituximab, Eculizumab) as “treatment-refractory” in accordance with current definitions[29].

### Standardized scores

To further assess the burden of disease, standardized scores were integrated in the questionnaire, (SF-36 (Short Form Health, i.e. general HRQoL) [33, 48], MG-Qol15 (Myasthenia gravis quality of life, i.e. MG-specific HRQoL) [7], MG-ADL (Myasthenia gravis activities of daily living profile) [49], CFQ-11 (Chalder Fatigue scale) [8, 20, 30], ESSI-D (ENRICHED Social Support Inventory) [19, 25] and HADS-D (Hospital anxiety and depression scale) [3, 15, 52]). In the SF-36 (0–100-point scale) and the ESSI-D (5–25-point scale), the higher the score, the better is the patients’ situation. Whereas in the MG-Qol15 (0–60-point scale), the MG-ADL (0–24-point scale), the HADS-D (0–21-point scale for each sub-scale anxiety and depression) and the CFQ11 (0–33-point scale) a high score indicates a worse situation. Additional to the Likert format’, the CFQ11 offers a binary scoring where 4 points or more equate severe fatigue [8]. In the ESSI-D, low social support is defined as a sum score of 18 or less and at least two items with 3 or less points [19]. With an HADS-D sub-score, participants scoring 8 points or more are defined as having substantial grades of anxiety or depression [3].

#### Imputation of missing values using the SF-36

Following the instructions of Morfeld et al. [33] to calculate the subscale scores of the SF-36, missing values were replaced by the mean values of the existing items of the same subscales, if at least 50% of the items were answered. For number of missing values with and without imputation of all subscales, see Supplement 1.

### Matched controls

To directly compare HRQoL to the general population (genP), we used data from participants of a German-wide representative study [24] (German Health Interview and Examination Survey for Adults, DEGS1, 2008–2011) which was conducted by the Robert Koch Institute aimed to repeatedly collect representative data on the health status, health-related behaviour, healthcare and living conditions of adults residing in Germany who are aged 18 and over. Information on gender and age was used for the matching of cases (MG patients) and controls using exact matching by gender, and matching by age groups (18–24) (25–29) (30–39) (40–49) (50–59) (60–69) in a ratio of 1:2. Due to the low number of possible controls in the age group 70 + years, matching for this age group was conducted in a ratio of 1:1, resulting in 1649 cases assigned to 2556 controls.

### Sociodemographic variables

Educational status was graded into three groups (low, medium, high) on the basis of information on the highest level of education according to the CASMIN classification [28]. Information of net household income was based on income categories: "Less than 1000€", "Between 1000€ and 2499€", "Between 2500 and 5000€" and "More than 5000€". For comparison with the control group, currency-equivalent values were assigned to these categorical responses (750 = less than 1000€; 1750 = 1000–2499€; 3750 = 2500–5000€; 5500 = more than 5000€). Net household income was weighed according to the number of people living in the household using the new OECD-modified scale [13].

For comparison with the DEGS1 sample, only income scores (and no data in euros) were available [27]. The calculated income values of the myasthenia sample were therefore assigned to the corresponding scores and summarized in three income groups (low = up to 1188 euro, medium = 1189–1833 euro, high = 1834 euro and more).

### Statistical analysis

The statistical calculations were performed using IBM Corp. Released 2017. IBM SPSS Statistics for Windows, Version 25.0. Armonk, NY: IBM Corp. using IBM SPSS Statistics 25 and R (version 3.5.3) [40] software. Net diagrams were created using Excel (version 2002) from Microsoft Office 365 ProPlus.

Depending on the scale and distribution of the outcome variables, appropriate descriptive statistics (mean, standard deviation, median, interquartile range, absolute and relative frequencies) are presented. Furthermore, parametric and non-parametric measures were used to test for group differences. A two-sided significance level of *α* = 0.05 was used. No adjustment for multiple testing was applied in this exploratory study. Linear mixed regression models adjusted for gender, age, educational status, income and partnership status were calculated (random intercept models, random intercept for matching ID) for the analyses of the differences between MG patients and controls in the SF-36 subdomains *physical functioning* and *emotional well-being*. Furthermore, interactions between disease status (MG: yes/no) and age, or sex were included. The multivariable analysis was carried out in the full analysis set including estimated values in case of missings. Multiple imputation (*m* = 10 datasets) was used to estimate missing using predictive mean matching and chained equations x complete datasets were created and separately analysed. The results were then combined using Rubin’s rules [42].

#### Net diagrams

To present various aspects of the burden of disease holistically in net diagrams, the different score values of MG-ADL, MG-QoL15, HADS, ESSI-D, CFQ11 and SF-36 subdomains were levelled on a unidirectional scale from zero (no complaints) to 100 points (strongest restrictions).

### Data availability

Data not provided in the article because of space limitations may be shared (anonymized) at the request of any qualified investigator for purposes of replicating procedures and results.

## Results

### Response analysis

Of the 3262 contacted members of the German Myasthenia Association (DMG), 103 persons were excluded retrospectively from response analysis, because they did not meet the inclusion criteria (e.g. diagnosis of Lambert Eaton myasthenic syndrome). The overall response rate was 52.5% (*n* = 1660). The age distribution of study participants (SP) is shown in Supplement 2.

### Patient characteristics

The occurrence of first MG symptoms was at the mean age of 49.3 (SD 19.7) years, with earlier start of symptoms in women (41.8, SD 19.6) than in men (58.6, SD 15.3). It took on average more than two years from the appearance of the first symptoms to diagnosis. The mean disease duration since diagnosis was 13.6 (SD 11.6) years (Supplement 3).

**Fig. 1:**
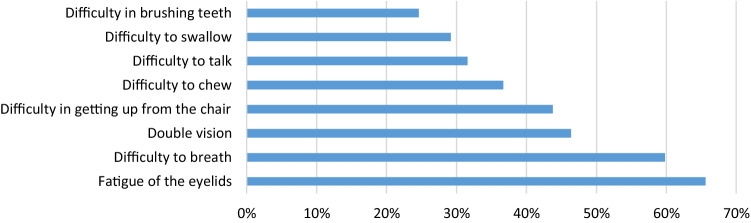
Symptoms of MG Patients according to single-item responses in the MG-ADL score (Activities of daily living). Symptoms were counted as presented if responses other than “normal” were selected by the study participants

Overall, 45.7% estimated the severity of MG as mild, 45.3% as moderate and 9% as severe (Table 1), with a substantial difference between men and women: Women reported more often a medium and high disease severity. More than one-fifth (21.3%) of the study participants (SP) were affected by an ocular subtype, 69.6% reported a generalized subtype. Within the subgroup of generalized MG (*n* = 1127), 42.0% reported impairment of swallowing and breathing and 48.0% reported impairment of predominantly limb muscles (Table 1); 228 (15.6%) of the SP met the criteria for treatment-refractory patients (supplement 3). In the MG-ADL, the median sum score was 4. Among the most frequent indicated sub-items of the MG-ADL were difficulty to breathe (59.8%) and double vision (46.4%) (further details, see Table 1 and Fig. 1). Symptoms were counted as presented if responses other than “normal” were selected by the SP. More than half (51.7%) of the SP reported acetylcholine receptor-antibodies (Ach-R-Abs), 4.9% Musk-ab and 14.6% reported to be seronegative. Almost one-third claimed not to know their antibody status. More than three-quarter (78.7%) of all SP reported at least one comorbid disease with cardiovascular diseases being the most common one (37%) followed by other autoimmune diseases (23.7%); 18.9% reported three or more comorbid diseases (Supplement 3).

**Table 1:**
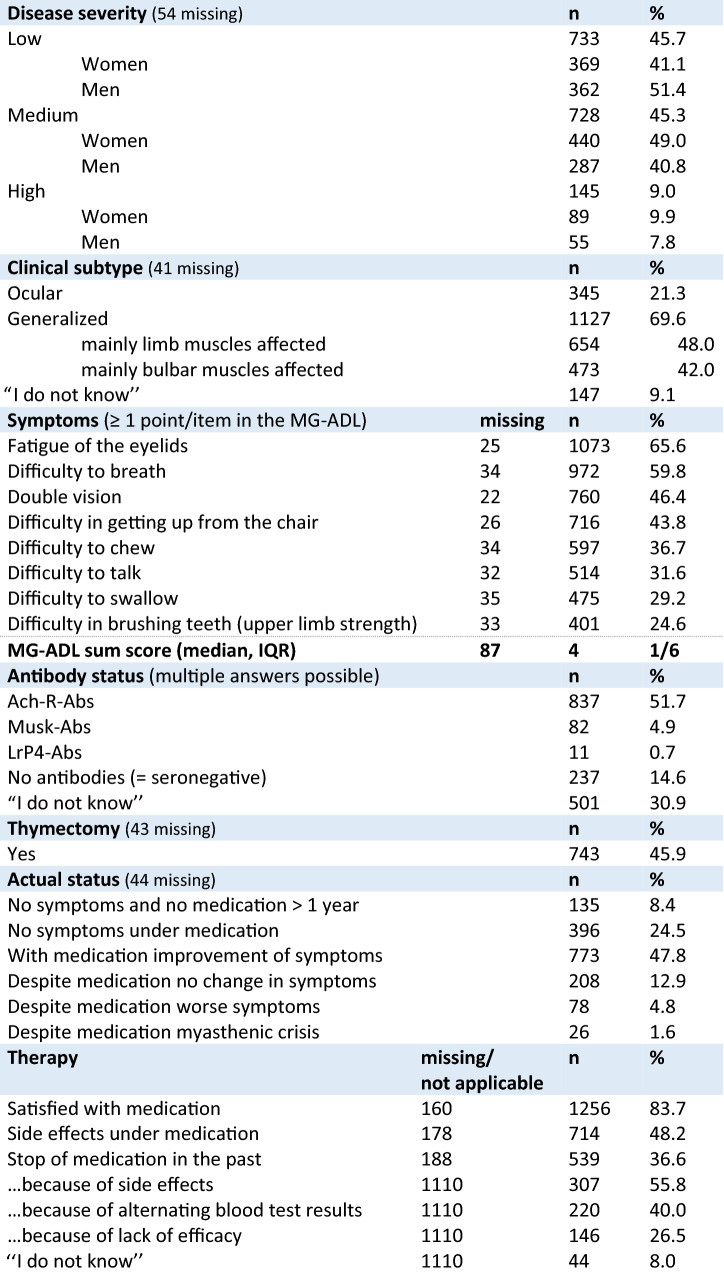
Clinical characteristics of study participants

Less than half (45.9%) of SP (*n* = 743) had undergone thymectomy (Table 1). Symptomatic treatment with pyridostigmine or pyridostigmine sustained release used 71.1% and 42.3% of all SP, respectively. Steroids (mean dosage of 6 mg/d) were used by 25.7%. Among the steroid-sparing immuno-suppressants, azathioprine was the most commonly used (45.5%) followed by mycophenolatmofetil (12.7%). Treatment was escalated with rituximab (6.2%), eculizumab (0.7%), IVIG (15.9%) and with PE or IA (7.2%) (Supplement 4).

Painkillers were used by 13.7%  of SP regularly and 9.5% took antidepressants (supplement 3). Asking for therapy response, 8.4% of SP reported no intake of medication and no symptoms since more than 1 year, corresponding to *complete stable remission* according the MGFA post-intervention status [22]. Almost one-quarter of SP (24.5%) reported *pharmacologic remission* (no symptoms under medication), whereas 47.8% stated *minimal manifestations* (symptoms under medication, although medication improves symptoms). 12.9% reported to have *unchanged status* (i.e. no change in symptoms under medication) and 4.8% reported *worse status*. Overall, 83.7% of SP are satisfied with their current medication (Table 1). Of all SP, 48.2% stated to experience current side effects under medication; 36.6% reported stop of medication or due to side effects (55.8%) or due to abnormal laboratory findings (40%) or due to lack of efficacy (26.5%) (Table 1, multiple answers possible).

Of all SP, 86% were living in a partnership (Table 2). In SP, who were separated or divorced, MG played a medium (14%) or high (14%) importance in the reason for separation. MG has influenced the family planning in16.8% (Table 2). Before having experienced first symptoms of MG, half of the SP (50.7%) were in full-time employment and 10.7% in part-time employment (Table 2). Formerly working patients were asked if they had experienced limitations regarding employment due to MG; this was affirmed by 72.6% of SP. In detail, 45.8% of them were incapable of working, 18.6% reported recurrent occupational disability, 12.7% had to reduce working hours, 7.8% could not work in the same profession anymore (professional disability) and 2.2% reported unemployment (63 patients selected multiple answers, Table 2). Almost two-thirds of the SP had a disabled person’s card (62.6%) with a median degree of disability of 60 (IQR 50–80), consistent with a moderate to severe degree of disability.

The majority of SP (46.3%) had an unweighted net household income between EUR 2500 and EUR 5000 per month (further details, Table 2). Being afraid of old-age poverty was affirmed by 486 (29.8%) respondents, among them 66.8% traced this fear back to MG.

**Table 2:**
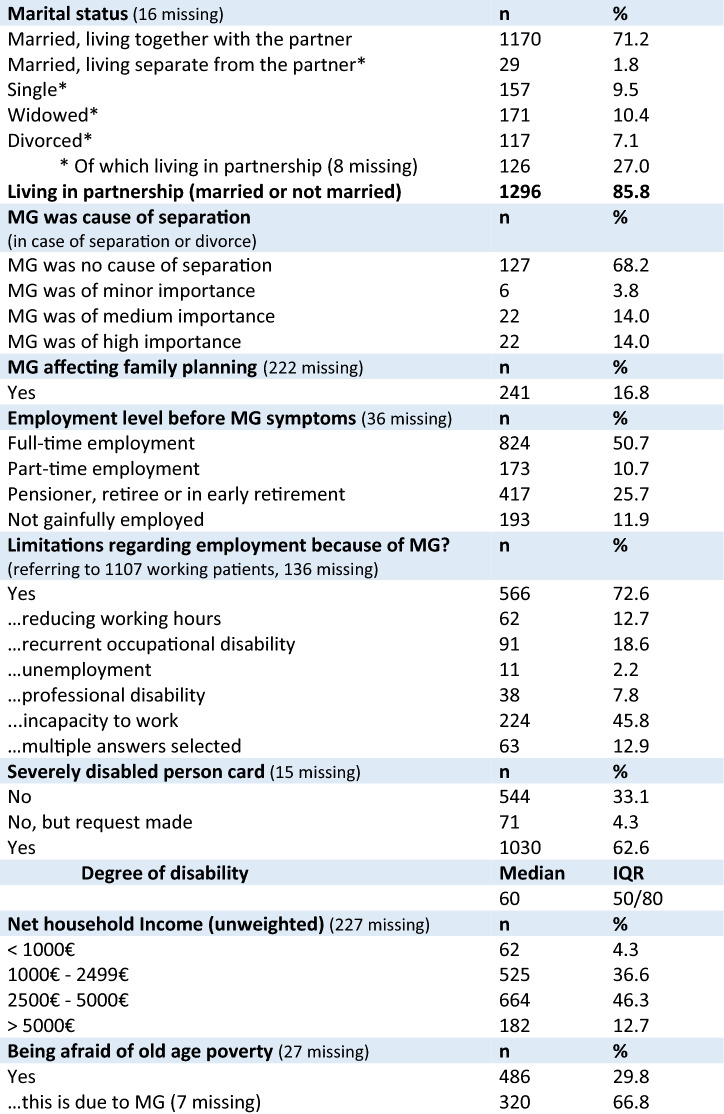
Sociodemographic characteristics of study participants

### Lower HRQoL (SF-36) of MG patients: a matched-pair comparison with the German general population (genP)

To analyse the health-related quality of life, we calculated a matched-pair comparison with the genP (control group) using data from the DEGS1 study. The education level of our patient population was higher compared to the control group (supplement 5). More SP (51.1%) were in the high-income group compared to the control group (42.4%), while more participants of the control group (34.4% vs. 19.7%) were in the medium-income group. The proportion of persons with low income was similar in both groups (23.2% in control group vs. 29.2% in SP).

**Fig. 2:**
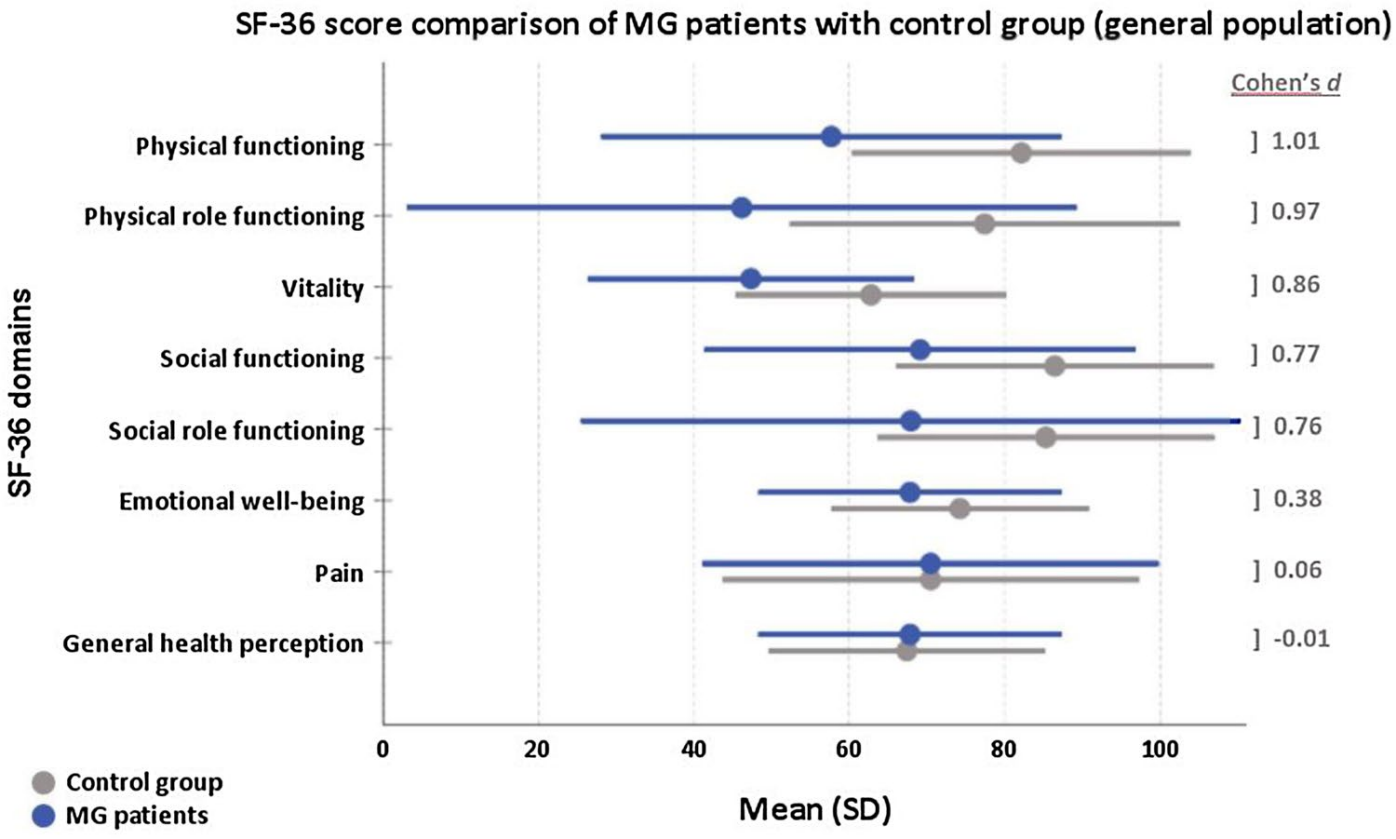
SF-36 domains: Mean values (and standard deviation, SD) of SP (=MG patients in blue) and control group (grey). A Cohen’s *d* > 0.5 indicates a high effect, 0.3–0.5 medium effect, 0.1–0.3 low effect and < 0.1 no effect

Figure 2 presents mean values of each of the eight domains of the SF-36. Apart from the domain, *General health perception* and *Pain* with no difference between the groups all other mean values of MG patients were lower compared to the control group with high statistical effect for the domains *physical functioning*, *physical role functioning*, *vitality* and medium effect for *social functioning* and *social role functioning* and low effect for the domain *emotional well-being*.

### Worse physical functioning (SF-36) in MG patients compared to general population

In multivariable analyses, MG patients showed a lower level of 25 (95% CI 22–29) points in *physical functioning* compared to the genP (linear mixed regression models adjusted for gender, age, educational status, income and partnership status, Table 3). Difference between genP and MG varied by age group with highest difference in 25–29y (32, 95% CI 16–48) and lowest difference in youngest age group 18–24y (18, 95% CI 3–33). In both groups, women reported lower values of *physical functioning* than men did. However, difference between genP and MG in males was 23 (95%CI 19–27), but 27 (95% CI 24–31) in women. Further similar associations of income, education and partnership status with physical functioning were present in both groups: Lower income, low and medium education and having no partner were associated with lower levels of physical functioning compared to the particular reference group.

**Table 3:**
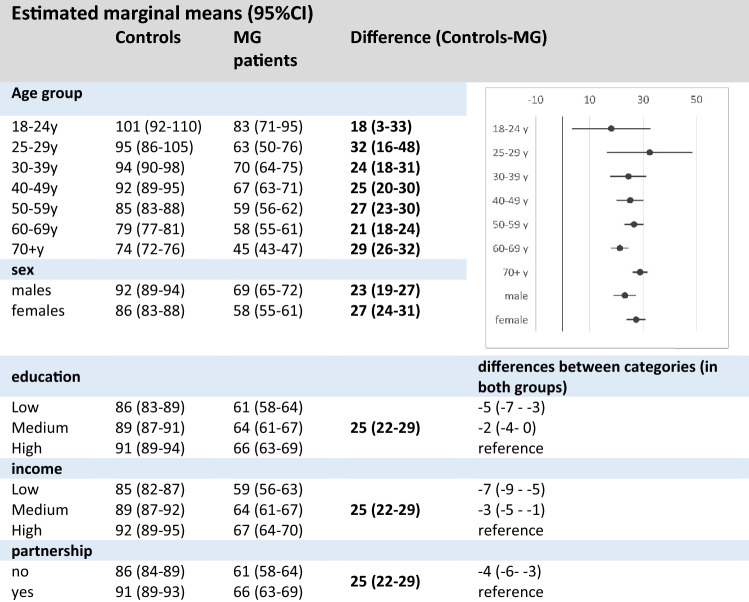
Multivariable analysis on physical functioning (SF-36) (combined results after multiple imputation, *n* = 4205)

(marginal means and 95% CI, model included interaction effect for group*sex and group*age group)

### Worse emotional well-being (SF-36) in MG patients compared to general population

In multivariable analyses of the SF-36 domain *emotional well-being*, MG patients reported lower values than genP (mean difference. 5 points, 95% CI 2–8 points). However, the differences were very variable depending on the age group (supplement 6). Comparing *emotional well-being* of MG with genP, the difference was substantial in 30 + years with largest differences in those 60 years and older. The emotional well-being of respondents with lower education as well as low income was lower compared to those with higher education and high income. Singles were more burdened than those in partnership.

### Myasthenia gravis specific scores and burden of disease

Compared to men, women reported higher levels of difficulties in activities of daily living (MG-ADL), lower MG-QoL15-scores, more symptoms of anxiety and depression (HADS), and of fatigue (CFQ11) as well as less perceived social support (Table 4). Patients with treatment-refractory MG demonstrate considerably worse scores in the MG-ADL, MG-QoL15, HADS and CFQ compared to the non-refractory. Musk-Abs-positive patients show higher scores (MG-ADL, MG-QoL15, HADS, CFQ) than AChR-Abs-positive patients (Table 4). Patients who had undergone thymectomy reported less difficulties in activities of daily living, less signs of fatigue and a better MG-QoL15-score than patients without thymectomy. There were no substantial differences between EOMG and LOMG.

**Table 4:** Overview MG-ADL, MG-QoL15, HADS, CFQ, ESSI-D and subgroups

Parameter	All	Men	Women	gMG	oMG	Refractory MG	Ach-R-Abs pos MG	Musk-Abs pos MG	Thym-ectomy	EOMG	LOMG
*n* (missing) %	1660 (0) 100	725 (4) 43.8	931 (4) 56.2	1127 (41) 69.6	345 (41) 21.3	228 (200) 15.6	837 (41) 51.7	82 (41) 5.1	743 (43) 45.9	176 (11) 10.7	1473 (11) 89.3
MG-ADL Median (IQR)	4 (1/6)	3 (1/5)	4 (7/12)	4 (2/7)	2 (1/4)	7 (4/10)	3 (1/6)	5 (2.25/7)	3 (1/6)	3 (1/6)	4 (2/6)
*p* values	–	**< 0.00**		**< 0.00**	**< 0.00**	**< 0.00**	**< 0.00**	**0.009**	**0.047**	0.292	
MG-QoL15 Median (IQR)	12 (4/25)	9 (3/21)	15 (5/28)	16 (5/28)	6 (2/14)	29 (19/38)	11 (3/23)	16 (8/25)	11 (3/24)	12 (3/28)	12 (4/24)
*p* values	–	**< 0.00**		**< 0.00**	**< 0.00**	**< 0.00**	**< 0.00**	0.072	**0.018**	0.523	
HADS Median (IQR)	10 (5/17)	9 (4/15)	11 (6/18)	11 (6/18)	8 (4/13)	12 (8/19)	9 (5/15)	10 (5/18)	9 (5/17)	10 (5/17)	10 (5/17)
*p* values	–	**< 0.00**		**< 0.00**	**< 0.00**	**< 0.00**	**0.018**	0.313	0.119	0.995	
HADS-A ≥ 8 p *n* (%)	520 (32.5)	179 (25.5)	339 (37.9)	393 (36.1)	78 (23.2)	86 (38.4)	234 (28.4)	26 (33.8)	229 (31.6)	64 (37.2)	452 (31.9)
HADS-D ≥ 8 p *n* (%)	446 (27.9)	184 (26.1)	260 (29.2)	347 (31.9)	57 (16.8)	84 (36.8)	191 (23.4)	24 (30.8)	177 (24.5)	40 (23.1)	402 (28.4)
CFQ sum (Likert) Median (IQR)	17 (12/21)	16 (12/21)	17 (12/22)	18 (13/22)	14 (11/18)	21 (17/25)	16 (12/21)	19 (13/23)	16 (12/21)	16 (12/21)	17 (12/22)
*p* values	–	**0.002**		**< 0.00**	**< 0.00**	**< 0.00**	**< 0.00**	**0.029**	**0.002**	0.187	
CFQ ≥ 4 (Binary) *n* (%)	989 (66.7)	426 (63.3)	560 (69.4)	728 (72.5)	161 (50.8)	183 (80.3)	483 (63.9)	55 (71.4)	424 (63.6)	103 (63.2)	880 (67.1)
ESSI-D ≤ 18 p *n* (%)	343 (22.7)	102 (15.9)	241 (27.9)	253 (24.4)	55 (17.6)	60 (26.3)	156 (20.2)	9 (11.8)	151 (21.8)	42 (24.0)	300 (22.6)
*p* values	–	**< 0.00**		**0.041**	**0.041**	0.122	**0.045**	**0.043**	0.503	0.687	

*gMG* generalized myasthenia gravis, *oMG* Ocular MG, *EOMG* Early Onset Myasthenia Gravis (≤ 45 years old), *LOMG* Late onset myasthenia gravis (> 45 years old), *MG-Qol15* Myasthenia gravis quality of life, *MG-ADL* Myasthenia gravis activities of daily living profile, *CFQ-11* Chalder Fatigue scale, *ESSI-D* ENRICHED Social Support Inventory, *HADS-D* Hospital anxiety and depression scale; p values indicate significance level or between men and women, or EOMG and LOMG or otherwise in the specific domain (e.g. gMG or Thymectomy) yes/no

significant *p* values (<0.05) are printed in bold

The MG-ADL and MG-QoL15 were positively correlated (Spearman’s correlation coefficient *r* = 0.77): The more difficulties of daily living have been reported, the lower was the HRQoL measured by MG-QoL15. A longer disease duration was correlated with a lower MG-QoL15 sum score suggesting a better HRQoL compared to persons affected by a shorter duration of MG (Spearman’s correlation coefficient *r* = − 0.96). In the HADS anxiety subscale, almost one-third (32.5%) showed 8 points or more, defined as presence of anxiety (Table 4). In the depression subscale, we found 12.7% of SP with signs of mild depression (8–10 points), 11.4% of SP with severe (11–14 points) and 3.8% of SP with signs of very severe depression (15–21 points). Patients with low social support (≤ 18 points in the ESSI-D) had more difficulties in daily life activities (median MG-ADL 5, IQR 3/8); they showed more symptoms of anxiety and depression (median HADS 16, IQR 10/22) and experienced a lower quality of life (median MG-QoL15 21, IQR 10/34) compared to patients with higher levels of social support (median MG-ADL 3, IQR 1/6, median HADS 9, IQR 4/14, median MG-QoL15 10.5, IQR 3/22).

**Fig. 3:**
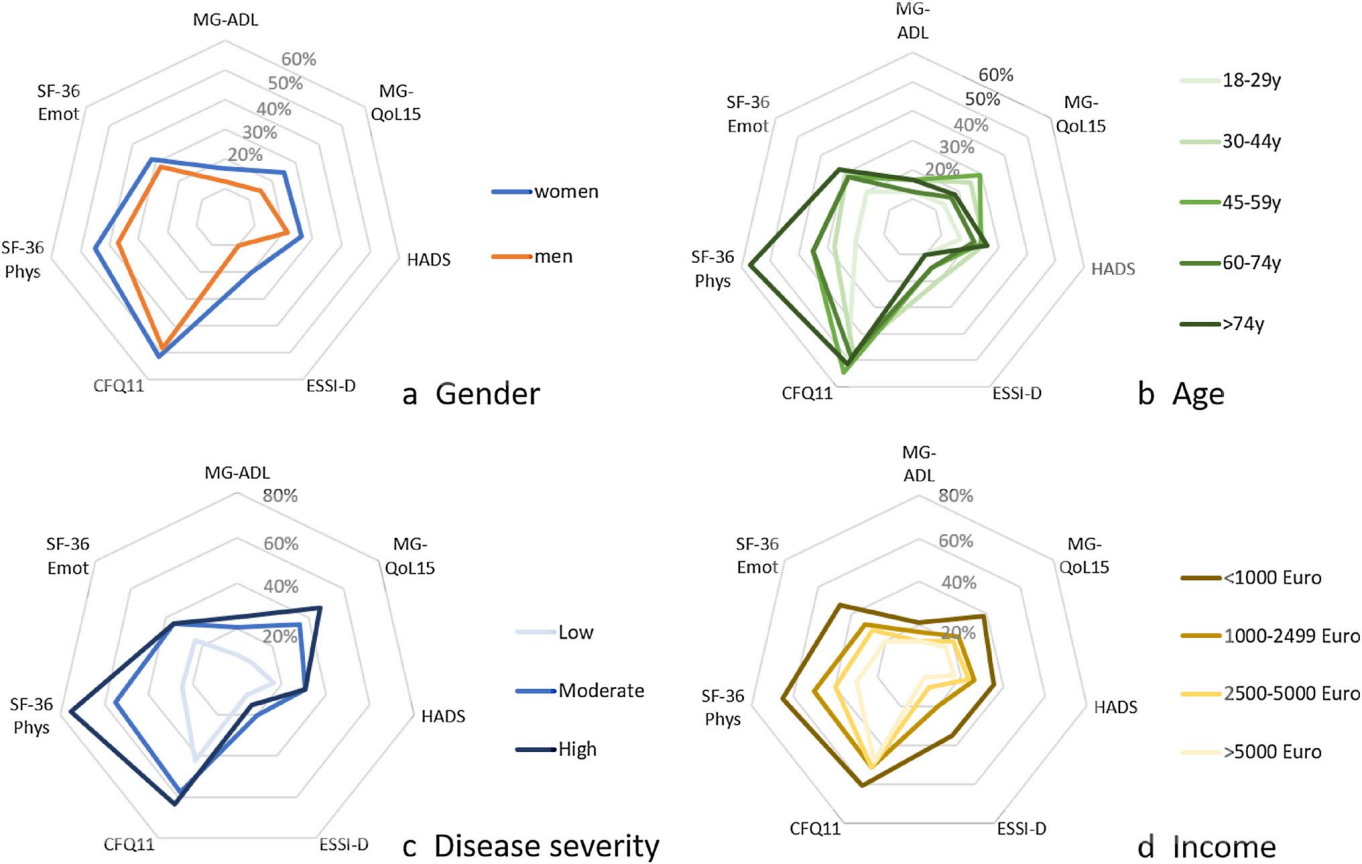
Net diagrams integrating the Myasthenia gravis Activities of Daily Living Score (MG-ADL), the Myasthenia gravis Quality of life Score (MG-QoL15), the Hospital Anxiety and Depression Scale (HADS), the ENRICHD Social Support Inventory (ESSI-D), the Myasthenia gravis Quality of life Score (MG-QoL15), the Hospital Anxiety and Depression Scale (HADS), the ENRICHD Social Support Inventory (ESSI-D), the Chalder Fatigue Scale (CFQ11) and the Physical Functioning (SF-36 Phys) and Emotional wellbeing (SF-36 Emot) domain of the Short Form 36 (SF-36) in different subgroups: **a** Gender, **b** age groups, **c** groups of different disease severity and d. net household income groups. The further out the lines are in the net, the higher and worse the single score value: Women (**a**), old patients (**b**), patients with high disease severity (**c**) and low income (**d**) do have the highest burden of disease, composed of high single score value

The individual aspects of the burden of MG as captured by the individual assessments were summarized in net diagrams (Fig. 3). Overall, the burden is higher in female patients, in patients with high disease severity levels, with low income and in middle-aged (and older) patients. Fatigue is present independent from age, gender or income, but fatigue is associated with disease severity.

## Discussion

In this study, we demonstrate that HRQoL is markedly lower in MG patients compared with the general population (genP). The overall burden is particularly high among women, in high disease severity levels, in low-income groups and among middle-aged and older patients.

Several studies have described a lower quality of life in MG patients with MG-specific and non-specific scores [5, 37, 38, 44]. The SF-36 has been used to compare means of patients’ values to normative values of controls [38]. In our in-depth analysis using a matched-pair comparison to the genP in Germany, large differences in the domains *physical functioning*, *physical role functioning* and *vitality* indicate a high individual burden for MG patients. Corresponding to our results, other studies [2, 5, 51] describe *physical role functioning*, *general health perception* and *vitality* as the domains with the lowest mean values. Interestingly, in our study, mean values in the domain *general health perception* (67.3, SD 19.7) do not differ from the genP (67.2, SD 17.9). Similar in the domain *pain*, no substantial difference to the genP was seen. Twork et al. conducted a large study with 1459 patients of the German Myasthenia Association (DMG) to explore quality of life [44]. Compared to these results published in 2010, mean values of the single SF-36 domains have not changed remarkably apart from *pain* (46.0[44] vs. 68.4 in our cohort) and *general health perception* (44.8[44] vs. 67.3). These two categories have now reached genP values, as mentioned above. In MG patients, effects of age on domains, such as *physical functioning* and *emotional well-being*, are much higher than in the genP. Income and education influence HRQoL in MG patients. However, with our novel matched-pair analysis, we can demonstrate that there are no major differences of these effects compared to the genP.

Compared to other chronic diseases that directly or indirectly impair muscle activity, e.g. rheumatoid arthritis (RA), some similarities can be observed, such as lower physical functioning (SF-36) in older patients compared to genP [32]. However, the positive association between mean age and the mental health domain described in RA cannot be observed in MG. Interestingly, comparison with other diseases that permanently impair control of muscle function also shows that performance in various SF-36 domains differs; for example, patients with Parkinson's disease and multiple sclerosis have similar limitations in the domain physical functioning as compared to MG. However, these two diseases show significantly greater differences in social role functioning and emotional well-being compared to the normal population than we observed in our cohort [41]. The extent to which disease-specific patterns can be derived from the SF-36 profiles needs to be investigated in comparative studies. Beyond lower HRQoL, we integrated further standardised scores in our analysis to draw a comprehensive picture of the individual burden of disease, among them the scores of anxiety and depression (HADS-D), fatigue (CFQ11) and social support (ESSI-D). The frequencies of anxiety and depression in MG are remarkable. Although these psychiatric comorbidities are similarly common in other chronic neurological diseases, such as Parkinson’s disease or multiple sclerosis, they should be considered in the treatment of patients as they are known to severely affect the well-being of those affected. As known from other chronic diseases like multiple sclerosis, psychiatric abnormalities essentially change self-perceived severity of disease, as well the perception of therapy response and success [26]. In our study, the proportion of SP with abnormal depression scale scores is highest in treatment-refractory patients. Further studies have to be conducted to evaluate the effect of depression on self-perceived severity and quality of life. Furthermore, nearly 60% reported persistent fatigue known to have a high impact on quality of life [17] in patients with MG. Low social support was reported by more than one-fifth of our study participants. Perceived social support, however, engages a health-promoting lifestyle [21] and in an Italian study (*n* = 74) perception of support is a predictor of mental health [39]. Therefore, low social support might increase the burden of disease. Clinical aspects, such as muscle weakness, double vision, myasthenic crisis, pain, sleep disturbances, the use of immunosuppressive drugs, and medication side effects, as well as demographic aspects, like gender, age, place of residence and medical infrastructure have been demonstrated to be additionally associated with a poor quality of life in MG patients [2, 5, 45] and thus influencing the burden of disease.

We paid special attention to MG influence on partnership and family planning, education level, employment situation and income as we suspect these aspects to have a high impact on the perceived burden of disease. A high percentage of the patients was living in partnership (86%), which is comparable to previous findings [6, 50]. One-third of patients require their partner to be their primary carer [9]. In nearly one-third of study participants, MG played a role in separation or divorce from a partner and in 16.8% MG influenced family planning. A large survey on 801 women with MG revealed that over fifty percent had abstained from having children due to MG [35], even if according to current knowledge, MG patients should not be discouraged from giving birth. Corresponding to findings in the literature [14], the age peak around 30–40 years of our cohort concerns mostly women [10]. This means that the first and initially often strong symptoms occur when patients still work and especially for young women this regards a period, when family planning and career building is an important topic.

A majority of our patients experienced limitations regarding employment due to MG such as incapacity of work or recurrent occupational disability. Similar, in an Italian cohort, at least two out of three MG patients suffered from changes in work and/or income [47] and a large Japanese cross-sectional study demonstrated that MG patients often experience unemployment (27.2%), involuntary job transfers (4.1%) and a decrease in income (35.9%) [34]. In an Australian cohort, 39.4% had stopped work due to MG and 19.4% had to change occupation [4]. Matched Danish MG patients experienced poorer labor market experience and suffered more often from long-term sick leave [11]. Our data demonstrate a negative influence of low income on the HRQoL in SF-36 sub-domains, such as physical functioning and emotional well-being.

So far, our study with 1660 participants is the largest conducted on this topic. Gender distribution is very comparable to our outpatient clinic (iMZ) and to other study groups from literature [46]. However, the population of the German Myasthenia Association (DMG) might not fully represent the “average German MG-patient”; e.g., this population is slightly older than the common MG-population [5, 6, 23, 51]. In addition, it is conceivable that more severely than mildly affected MG patients might register as members of a patient organization like the DMG. In addition, we cannot rule out selection bias: Members of the German self-help organization might have a higher educational level than MG patients who do not register themselves in a patient organisation. Because the questionnaire was written in German and asked specific aspects about the disease, MG patients whose native language is not German or less educated patients might not have returned the questionnaire. Eventually only highly motivated and less sick patients completed the questionnaire. For this reason, we offered a long response time of 4 months to catch moments when patients felt able to fill out the questionnaire. When asking for information that lies far in the past like age of symptom onset, a recall bias could have affected the results. However, the majority of questions was related to the current situation and recall bias should be small, but even relevant and an explanation for some missing answers. Another weakness of our study is that the data of the comparison group (genP) [24] were collected 10 years ago and some answers might have changed over time. Using a questionnaire which was sent back anonymously did not allow to compare and validate the statements of patients with clinical data or to add objective data of examinations performed by a health care professional. Also, not every questionnaire was validated for use in German (but already used in research [17, 25, 30]) and, in case of the MG-ADL [49], not yet validated for independent completion by a patient (ongoing study in our research center). However, we know from other studies in which MG patients were both examined by a doctor in standardized tests and performed self-declarations that objective and subjective data correlate with each other [36, 46], with the restriction that the data collection was not anonymous in these studies but was carried out by investigators. The term “treatment-refractory MG” was used in accordance to current literature [18] even if "non-responders to standard treatments" or "high disease activity despite standard treatments" would be more appropriate. The strengths of our study are the matched-pair analysis, a comprehensive multidimensional approach, a representative cohort and a high number of participants (*n* = 1660, response rate 52.5%) offering a large dataset in the real-world setting.

## Conclusion

Our study emphasizes that the mental and physical health-related quality of life in MG patients is remarkably lower in comparison to the genP. Quality of life reflects one aspect of the burden of disease. Our data demonstrate that many factors are a piece of the puzzle to create a holistic view on the burden of disease. It would make sense to develop a tool that integrates other influencing factors besides quality of life, such as functional level, depression and anxiety, fatigue and social participation. In recent and current phase-III studies, disease-specific PROMS are the primary and secondary outcome measurements [18]. This highlights that the perceived subjective experience of the individual MG patient is the most relevant parameter to improve. Our data warrant the need to conduct prospective multicenter studies to assess the individual burden of disease including generic scores like the SF-36 to make results comparable with the normal population. Special attention should be paid to gender aspects as women suffering from MG do have a higher burden of disease.

## Supplementary Information

Below is the link to the electronic supplementary material.

Supplementary file1 (DOCX 132 KB)

## Data Availability

The study was conducted in accordance to the declaration of Helsinki and the STROBE reporting guidelines.
