# Correction to: Burden of disease in myasthenia gravis: taking the patient’s perspective

**DOI:** 10.1007/s00415-022-11290-w

**Published:** 2022-08-12

**Authors:** Sophie Lehnerer, Jonas Jacobi, Ralph Schilling, Ulrike Grittner, Derin Marbin, Lea Gerischer, Frauke Stascheit, Maike Krause, Sarah Hoffmann, Andreas Meisel

**Affiliations:** 1 Department of Neurology with Experimental Neurology, Charité University Medicine Berlin, Corporate Member of Freie Universität Berlin and Humboldt-Universität Zu Berlin, Charitéplatz 1, 10117 Berlin, Germany; 2 NeuroCure Clinical Research Center, Charité University Medicine Berlin, Charitéplatz 1, 10117 Berlin, Germany; 3 Center for Stroke Research Berlin, Charité University Medicine Berlin, Charitéplatz 1, 10117 Berlin, Germany; 4 Institute of Biometry and Clinical Epidemiology, Charité University Medicine Berlin, Charitéplatz 1, 10117 Berlin, Germany; 5 Institute for Social Medicine, Epidemiology and Health Economics, Charité University Medicine Berlin, Luisenstraße 57, 10117 Berlin, Germany; 6 Core Facility Genomics, Berlin Institute of Health at Charité University Medicine Berlin, Charitéplatz 1, 10117 Berlin, Germany; 7 Department of Psychiatry, Psychotherapy and Psychosomatics, Charité University Medicine Berlin at St. Hedwig Hospital, Große Hamburger Straße 5-11, 10115 Berlin, Germany

## Correction to: Journal of Neurology (2022) 269:3050–3063 10.1007/s00415-021-10891-1

The original version of this article unfortunately contained a mistake. The wrong name was given for a subgroup analysis.

The corrected details are given below for your reading.

The last sentence in the following section ‘’Myasthenia gravis specific scores and burden of disease’’ should read as

There were no substantial differences between patients younger or older than 45 years old at the time the study was performed.

Instead of EOMG (Early onset Myasthena gravis) and LOMG (Late onset Myasthenia gravis), the subgroups in Table 4 are “Patients ≤ 45 years old” and “Patients > 45 years old”, respectively.

The corrected Table 4 is given in the following page.

**Table 4:** Overview MG-ADL, MG-QoL15, HADS, CFQ, ESSI-D and subgroups

Parameter	All	Men	Women	gMG	oMG	Refractory MG	Ach-R-Abs pos MG	Musk-Abs pos MG	Thymectomy	≤ 45 years old	> 45 years old
***n*** (missing) %	1660 (0) 100	725 (4) 43.8	931 (4) 56.2	1127 (41) 69.6	345 (41) 21.3	228 (200) 15.6	837 (41) 51.7	82 (41) 5.1	743 (43) 45.9	176 (11) 10.7	1473 (11) 89.3
**MG-ADL** Median (IQR)	4 (1/6)	3 (1/5)	4 (7/12)	4 (2/7)	2 (1/4)	7 (4/10)	3 (1/6)	5 (2.25/7)	3 (1/6)	3 (1/6)	4 (2/6)
* p*-values	-	** < 0.00**		** < 0.00**	** < 0.00**	** < 0.00**	** < 0.00**	**0.009**	**0.047**	0.292	
**MG-QoL15** Median (IQR)	12 (4/25)	9 (3/21)	15 (5/28)	16 (5/28)	6 (2/14)	29 (19/38)	11 (3/23)	16 (8/25)	11 (3/24)	12 (3/28)	12 (4/24)
* p*-values	-	** < 0.00**		** < 0.00**	** < 0.00**	** < 0.00**	** < 0.00**	0.072	**0.018**	0.523	
**HADS** Median (IQR)	10 (5/17)	9 (4/15)	11 (6/18)	11 (6/18)	8 (4/13)	12 (8/19)	9 (5/15)	10 (5/18)	9 (5/17)	10 (5/17)	10 (5/17)
* p*-values	-	** < 0.00**		** < 0.00**	** < 0.00**	** < 0.00**	**0.018**	0.313	0.119	0.995	
**HADS-A** ≥ 8 p. *n* (%)	520 (32.5)	179 (25.5)	339 (37.9)	393 (36.1)	78 (23.2)	86 (38.4)	234 (28.4)	26 (33.8)	229 (31.6)	64 (37.2)	452 (31.9)
**HADS-D** ≥ 8 p. *n* (%)	446 (27.9)	184 (26.1)	260 (29.2)	347 (31.9)	57 (16.8)	84 (36.8)	191 (23.4)	24 (30.8)	177 (24.5)	40 (23.1)	402 (28.4)
**CFQ** sum (Likert) Median (IQR)	17 (12/21)	16 (12/21)	17 (12/22)	18 (13/22)	14 (11/18)	21 (17/25)	16 (12/21)	19 (13/23)	16 (12/21)	16 (12/21)	17 (12/22)
* p*-values	-	**0.002**		** < 0.00**	** < 0.00**	** < 0.00**	** < 0.00**	**0.029**	**0.002**	0.187	
**CFQ** ≥ 4 (Binary) *n* (%)	989 (66.7)	426 (63.3)	560 (69.4)	728 (72.5)	161 (50.8)	183 (80.3)	483 (63.9)	55 (71.4)	424 (63.6)	103 (63.2)	880 (67.1)
**ESSI-D** ≤ 18 p. *n* (%)	343 (22.7)	102 (15.9)	241 (27.9)	253 (24.4)	55 (17.6)	60 (26.3)	156 (20.2)	9 (11.8)	151 (21.8)	42 (24.0)	300 (22.6)
* p*-values	-	** < 0.00**		**0.041**	**0.041**	0.122	**0.045**	**0.043**	0.503	0.687	

gMG, generalized myasthenia gravis; oMG, ocular MG; MG-Qol15, myasthenia gravis quality of life; MG-ADL, myasthenia gravis activities of daily living profile; CFQ-11, Chalder Fatigue scale; ESSI-D, ENRICHED Social Support Inventory; HADS-D, Hospital anxiety and depression scale; *p*-values indicate significance level or between men and women, or patients ≤45 years old and >45 years old or otherwise in the specific domain (e.g. gMG or Thymectomy) yes/no

