# Supplementary material for: Burden of disease in myasthenia gravis: taking the patient’s perspective
Source: J Neurol. 2021 Nov 20;269(6):3050–63. doi: 10.1007/s00415-021-10891-1 (PMC9120127; doi:10.1007/s00415-021-10891-1)
Supplement: Supplementary file 1 — Supplementary file1 (DOCX 132 KB) [file 415_2021_10891_MOESM1_ESM.docx]

**Supplemental data**

**Burden of disease in myasthenia gravis – taking the patient’s perspective**

***Journal of Neurology***

Sophie Lehnerer*, Jonas Jacobi, Ralph Schilling, Ulrike Grittner, Derin Marbin, Lea Gerischer, Frauke Stascheit, Maike Krause, Sarah Hoffmann, Andreas Meisel

* corresponding author^1,2,3^ (Sophie.lehnerer@charite.de)

1 Charité – Universitätsmedizin Berlin, corporate member of Freie Universität Berlin and Humboldt-Universität zu Berlin, Department of Neurology with Experimental Neurology, Charitéplatz 1, 10117 Berlin, Germany

2 NeuroCure Clinical Research Center, Charité University Medicine Berlin, Charitéplatz 1, 10117 Berlin, Germany

3 Center for Stroke Research Berlin, Charité University Medicine Berlin, Charitéplatz 1, 10117 Berlin, Germany

Supplement 1 Number of missing values with and without imputation

| **Subscales of the SF-36** | **Number of missing values** | |
| --- | --- | --- |
|  | **without imputation** | **with imputation** |
| Physical functioning | 112 | 22 |
| Physical role functioning | 141 | 116 |
| Bodily pain | 56 | 25 |
| General health | 81 | 49 |
| Vitality | 76 | 31 |
| Social role functioning | 53 | 13 |
| Mental health | 81 | 38 |
| Emotional role functioning | 144 | 133 |
|  |  |  |

Supplement 2 **Age distribution** of study participants (SP), men (blue) and women (orange), 4 missings. The gender ratio of SP did not differ significantly from the entire group of DMG-members, all being invited to fill out the questionnaire (SD 0.00). Mean age of male


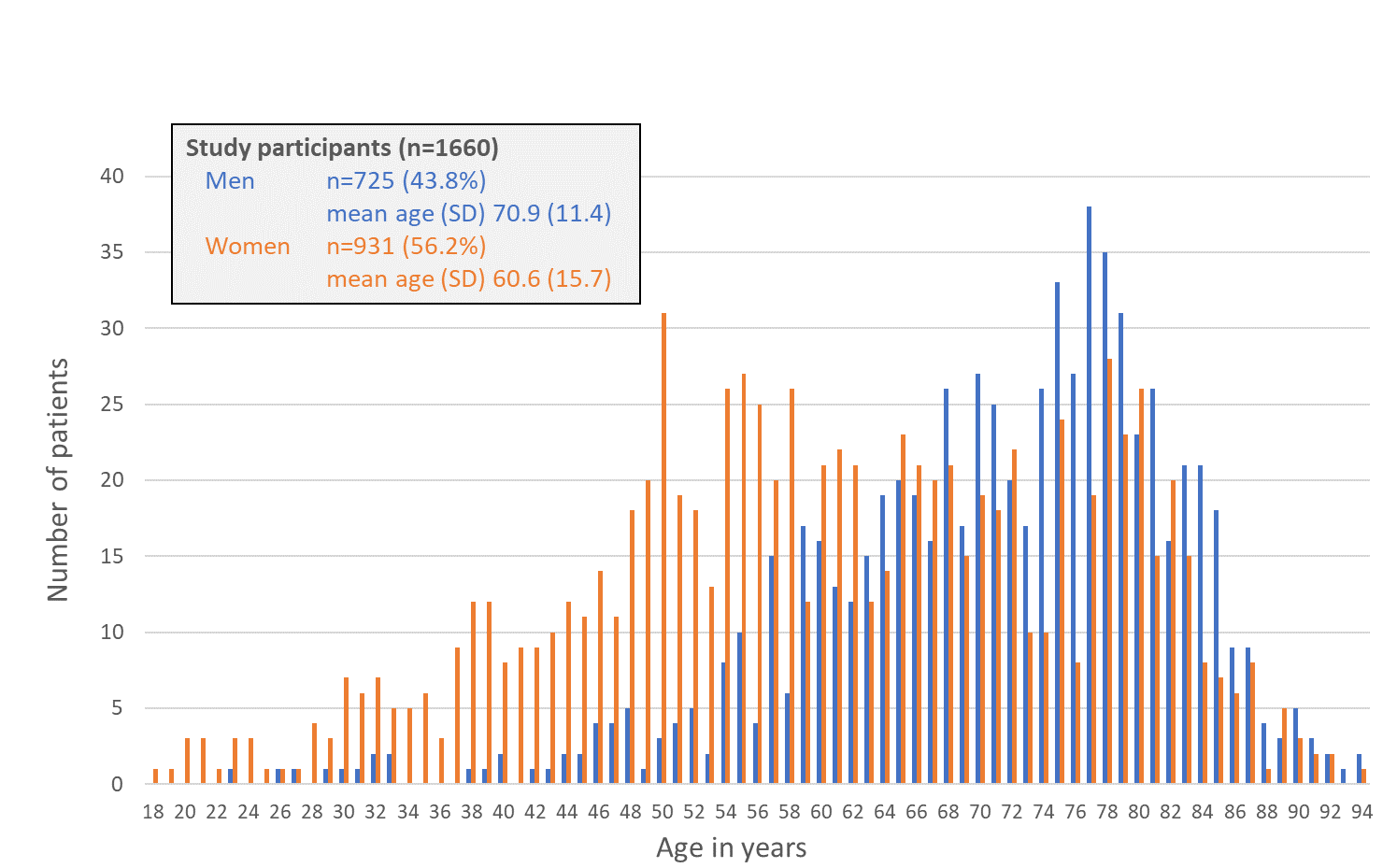


Supplement 3 Additional clinical characteristics of study participants

| **Age at onset of symptoms** (123 missing) | | |  | **Mean (SD)** |
| --- | --- | --- | --- | --- |
| Total (n=1537) | | |  | 49.3 (19.7) |
| Men (n=683) | | |  | 58.6 (15.3) |
| Women (n=850) | | |  | 41.8 (19.6) |
| **Duration from first symptoms to diagnosis (years)** (157 missing) | | | | **Mean (SD)** |
| Total (n=1503) | | |  | 2.14 (5.8) |
| Men (n=670) | | |  | 1.28 (4.8) |
| Women (n=829) | | |  | 2.83 (6.4) |
| **Disease Duration (since diagnosis)** (45 missing) | | | | **Mean (SD)** |
| Total (n=1615) | | |  | 13.6 (11.6) |
| Men (n=710) | | |  | 11.0 (9.4) |
| Women (n=905) | | |  | 15.6 (12.7) |
| **Categories of disease severity (subgroups)** | | **missing** | **n** | **%** |
| Generalized MG, medium or high disease severity | | 81 | 695 | 44.0 |
| …and with any exacerbation medication (Rituximab, Eculizumab, IVIG, PPH) | | 200 | 228 | 15.6 |
| **Comorbid diseases** | | | **n** | **%** |
| Cardiovascular disease | | | 614 | 37.0 |
| Autoimmune disease | | | 380 | 23.7 |
| Neurologic disease | | | 381 | 23.0 |
| Osteoporosis | | | 268 | 16.1 |
| Diabetes mellitus | | | 248 | 14.9 |
| Cancer | | | 222 | 13.4 |
| Lung disease | | | 186 | 11.2 |
| Psychiatric disorder | | | 121 | 7.3 |
| Other diseases | | | 463 | 25.9 |
| No comorbid disease | | | 354 | 21.3 |
| At least 1 comorbid disease | | | 1306 | 78.7 |
| At least 2 comorbid diseases | | | 704 | 42.2 |
| 3 and more comorbid diseases | | | 313 | 18.9 |
| **Co-medication** | **missing** | | **n** | **%** |
| Intake of antidepressants | 38 | | 154 | 9.5 |
| Intake of painkillers (regularly) | 38 | | 223 | 13.7 |

Supplement 4 Current medication

| **Current Medication** | **missing** | **n** | **%** | **% in the last 6 months** | **Dosage/d**  **Mean (mg) (STD)** | **missing (Dosage)** |
| --- | --- | --- | --- | --- | --- | --- |
| Pyridostigmine | 135 | 1085 | 71.1 |  | 180 (255.2) | 621 |
| Pyridostigmine sustained release | 136 | 644 | 42.3 |  | 180 (128.3) | 1033 |
| Mycophenolatmofetil | 136 | 194 | 12.7 |  | 1500 (684.6) | 1469 |
| Steroids | 136 | 391 | 25.7 |  | 5.25 (11.8) | 1280 |
| Azathioprine | 136 | 694 | 45.5 |  | 100 (64.9) | 988 |
| Methotrexate | 136 | 63 | 4.1 |  | 10 (18.8) | 1599 |
| Cyclosporine A | 136 | 14 | 0.9 |  | 150 (90.6) | 1647 |
| Rituximab | 135 | 94 | 6.2 | 53.6 |  |  |
| Eculizumab | 136 | 10 | 0.7 | 42.9 |  |  |
| IVIG | 134 | 243 | 15.9 | 42.2 |  |  |
| Plasmapheresis/Immunoabsorption | 135 | 110 | 7.2 | 13.4 |  |  |

Supplement 5 Matching MG-patients with general population (control group, data from DEGS1 study), distribution of gender, age, education and weighted income

|  | **Patients with MG** | | **Control group** | | |
| --- | --- | --- | --- | --- | --- |
|  | n=1649 | | n=2556 | | |
| **Gender** | **n** | **%** | **n** | **%** | |
| Men | 723 | 43.8 | 1004 | 39.3 | |
| Women | 926 | 56.2 | 1552 | 60.8 | |
| **Age** | **n** | **%** | **n** | **% (weighted %)** | |
| 18 - 24 years old | 16 | 1.0 | 32 | 1.3 (1.0) | |
| 25 - 29 years old | 13 | 0.8 | 26 | 1.0 (0.8) | |
| 30 - 39 years old | 80 | 4.9 | 160 | 6.3 (4.9) | |
| 40 - 49 years old | 144 | 8.7 | 288 | 11.3 (8.7) | |
| 50 - 59 years old | 291 | 17.6 | 582 | 22.8 (17.6) | |
| 60 - 69 years old | 363 | 22.0 | 726 | 28.4 (22.0) | |
| 70 and more years old | 742 | 45.0 | 742 | 29.0 (45.0) | |
| **Education (CASMIN)** | **n** | **%** | **n** | **%** |  |
| High | 481 | 29.5 | 500 | 19.9 | p<0.001 Cohen's d=0.248 |
| Medium | 701 | 43.0 | 1049 | 41.8 |  |
| Low | 449 | 27.5 | 962 | 38.3 |  |
| **Income (weighted)** | **n** | **%** | **n** | **%** |  |
| High | 734 | 51.1 | 592 | 42.4 | p<0.001 Cohen's d=0.446 |
| Medium | 283 | 19.7 | 880 | 34.4 |  |
| Low | 420 | 29.2 | 1084 | 23.2 |  |

Supplement 6 **Multivariable analysis on emotional well-being (SF-36)** (combined results after multiple imputation, n=4205). (marginal means and 95%CI, model included interaction effect for group*sex and group*age group)

| **Estimated marginal means (95%CI)** | | | | |
| --- | --- | --- | --- | --- |
|  | **Controls** | **MG patients** | **Difference (Controls-MG)** | |
| **Age group** |  |  |  | 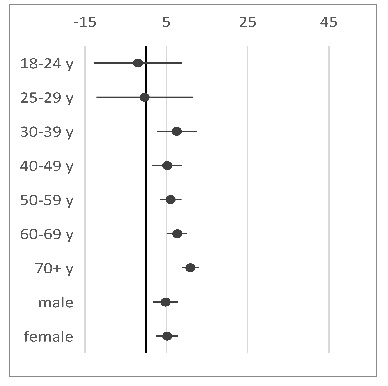 |
| 18-24y | 77 (71-83) | 79 (70-88) | **-2 (-13-9)** |  |
| 25-29y | 71 (64-78) | 71 (62-81) | **0 (-12-11)** |  |
| 30-39y | 74 (71-77) | 66 (62-70) | **8 (3-12)** |  |
| 40-49y | 74 (72-76) | 69 (66-72) | **5 (1-9)** |  |
| 50-59y | 72 (70-73) | 66 (64-68) | **6 (3-9)** |  |
| 60-69y | 74 (73-75) | 66 (64-68) | **8 (5-10)** |  |
| 70+y | 76 (74-77) | 65 (63-66) | **11 (9-13)** |  |
| **sex** |  |  |  |  |
| males | 76 (74-78) | 71 (69-74) | **5 (2-8)** |  |
| females | 72 (70-73) | 66 (64-69) | **5 (2-8)** |  |
| **education** |  |  |  | **differences between categories (in both groups)** |
| Low | 73 (71-75) | 68 (66-70) | **5 (2-8)** | -2 (-3 - 0) |
| Medium | 74 (72-76) | 69 (67-71) |  | -1 (-2- 1) |
| High | 75 (73-77) | 70 (67-72) |  | reference |
| **income** |  |  |  |  |
| Low | 72 (70-73) | 67 (64-69) | **5 (2-8)** | -4 (-6 - -3) |
| Medium | 74 (73-76) | 69 (67-72) |  | -1 (-3 - 0) |
| High | 76 (74-78) | 71 (68-73) |  | reference |
| **partnership** |  |  |  |  |
| no | 72 (70-74) | 67 (65-70) | **5 (2-8)** | -3 (-5- -2) |
| yes | 76 (74-77) | 71 (68-73) |  | reference |
